# Regional and network patterns of sleep-depth-associated brain diffusion changes: an EEG-informed multishell diffusion MRI study

**DOI:** 10.3389/fnins.2026.1889651

**Published:** 2026-09-15

**Authors:** Jehyeong Yeon, Chul-Ho Sohn, Chang-Soo Yun, Kun-Jin Chung, Byong-Ji Min, Chang-Ho Yun, Bong Soo Han

**Affiliations:** 1 Department of Radiation Convergence Engineering, College of Software and Digital Healthcare Convergence, Yonsei University, Wonju, Gangwon-State, Republic of Korea; 2 Department of Radiology, Seoul National University College of Medicine, Seoul National University Hospital, Seoul, Republic of Korea; 3 Neuroscience Research Institute, Gachon University Gil Medical Center, Incheon, Republic of Korea; 4 Department of Neurology, Seoul National University Bundang Hospital and Seoul National University College of Medicine, Seongnam, Republic of Korea

**Keywords:** diffusion kurtosis, diffusion MRI, EEG, glymphatic system, network, sleep

## Abstract

Sleep is thought to modulate brain fluid transport; however, whether these physiological processes are reflected in regional diffusion magnetic resonance imaging (MRI) parameters and their interregional organization during sleep remains unclear. We performed simultaneous electroencephalography (EEG) and repeated multishell diffusion MRI during zolpidem-facilitated sleep in 12 healthy adults. Analyses included nine participants who maintained sleep throughout all diffusion acquisitions. Scan-wise sleep depth was quantified using the odds ratio product (ORP), which showed excellent agreement with EEG-defined arousal states and an expected inverse association with relative delta power. The diffusion tensor and kurtosis metrics were quantified within the JHU ICBM-DTI-81 white-matter and the Automated Anatomical Labeling atlas, v. 2 (AAL2) gray-matter regions. We examined regional sleep-depth associations and interregional correlation networks. Exploratory analyses showed that deeper sleep was associated with lower white-matter anisotropy, reflected in reduced fractional anisotropy and kurtosis fractional anisotropy within major projection and association pathways. In the gray matter, associations with sleep depth were more pronounced, with lower axial, mean, and radial diffusivities, whereas the corresponding kurtosis metrics showed associations in the opposite direction that reached nominal significance. These associations were primarily localized to the frontocentral sensorimotor and medial frontal–cingulate regions. Correlation network analysis identified seven interregional correlations that were sensitive to sleep depth after controlling for global covariance. Four were located within the middle cerebral artery territory, two within the posterior circulation cerebellar and vermian regions, and one sensorimotor correlation spanned the anterior and middle cerebral artery territories. These findings demonstrate region-specific and coordinated diffusion changes during human sleep and establish an exploratory network-based framework for investigating diffusion patterns relevant to glymphatic physiology.

## 1 Introduction

Sleep supports synaptic homeostasis, metabolic regulation, and brain restoration (Cirelli and Tononi, 2008; Rasch and Born, 2013; de Vivo et al., 2017; Krause et al., 2017). The glymphatic system (GS) has been proposed as a brain-wide fluid transport pathway that promotes cerebrospinal fluid (CSF)–interstitial fluid (ISF) exchange and facilitates the clearance of soluble metabolites from the brain parenchyma (Iliff et al., 2012). This pathway is thought to operate along perivascular spaces formed in close association with astroglial endfeet, and its efficiency is influenced by aquaporin-4 (AQP4) water channels localized around cerebral vessels. Experimental studies in rodents have shown that natural sleep and anesthesia are associated with expansion of the extracellular space (ECS) and enhanced CSF–ISF exchange (Xie et al., 2013). These physiological changes have been linked to increased clearance of metabolites, including amyloid-β, suggesting a mechanistic connection between sleep, brain fluid transport, and neurotoxic waste removal (Peng et al., 2016; Nedergaard and Goldman, 2020).

Human neuroimaging studies have shown that sleep is accompanied by measurable changes in CSF dynamics, vascular physiology, and brain water diffusion. Simultaneous electroencephalography (EEG) and functional MRI (fMRI) recordings showed coordinated low-frequency neural and hemodynamic oscillations accompanied by retrograde CSF inflow into the fourth ventricle during non-rapid eye movement (NREM) sleep (Fultz et al., 2019). Other sleep MRI studies reported increased vasomotor, respiratory, and cardiac pulsations during NREM sleep and fluid pulsations related to autonomic arousals (Helakari et al., 2022; Picchioni et al., 2022). Recent evidence suggests that respiratory- and cardiac-driven brain pulsations become more pronounced during deeper NREM sleep and correlate with EEG delta power, providing further support for diffusion MRI as a potential marker of sleep-related brain water dynamics (Ulv Larsen et al., 2025). Diffusion MRI (dMRI) studies have also reported sleep-related changes in the apparent diffusion coefficient (ADC) and diffusion kurtosis parameters, suggesting that sleep can alter tissue water mobility and diffusion heterogeneity (Le Bihan et al., 1988; Basser et al., 1994; Demiral et al., 2019; Örzsik et al., 2023). However, these diffusion changes should be interpreted cautiously because dMRI does not directly measure glymphatic flow but rather provides indirect information about water mobility within tissue and fluid compartments.

Despite the growing interest in sleep-dependent glymphatic function, the direct and repeated assessment of glymphatic transport in the human brain remains technically challenging. Contrast-enhanced MRI can visualize CSF tracer movement and provide information about brain-wide clearance pathways; however, its invasive nature limits repeated measurements during natural sleep (Eide and Ringstad, 2015; Ringstad et al., 2018; Eide et al., 2022). As a non-invasive alternative, diffusion tensor imaging analysis along the perivascular space (DTI-ALPS) has been introduced as an indirect MRI-based index related to glymphatic or interstitial fluid dynamics (Taoka et al., 2017). DTI-ALPS estimates water diffusivity along the direction of the deep medullary perivascular spaces while reducing the influence of major white matter (WM) fiber tracts. This method has been applied in Alzheimer’s disease, obstructive sleep apnea, rapid eye movement sleep behavior disorder, and other conditions in which glymphatic dysfunction has been hypothesized (Taoka et al., 2017; Lee et al., 2022; Bae et al., 2023).

However, current DTI-ALPS-based approaches have important limitations. The majority of the studies have relied on single-time-point measurements, predefined anatomical locations, or summary indices, which may not fully capture sleep-related diffusion changes that evolve over time and vary across brain regions. Moreover, because DTI-ALPS is calculated from diffusion measurements in periventricular WM, it does not directly characterize sleep-related diffusion changes in gray matter (GM) or their distribution across the GM and WM. Moreover, recent reproducibility and methodological studies have shown that the ALPS index is affected by imaging parameters, scanner/protocol differences, ROI placement, anatomical assumptions, and analysis strategies (Taoka et al., 2022; Saito et al., 2023; Satpathi et al., 2025). Therefore, DTI-ALPS should be regarded as a diffusion-based proxy for perivascular-space diffusion in the WM rather than a direct measure of glymphatic transport, interstitial fluid flow, or solute clearance. These limitations indicate the need for complementary analytical frameworks that can characterize diffusion changes across both GM and WM and across repeated sleep measurements.

A key unresolved question is whether sleep-related tissue and fluid changes relevant to GS function occur uniformly across the brain or show region-specific temporal coordination with sleep depth. Sleep consists of physiological states characterized by different levels of neuronal synchronization, vascular regulation, autonomic tone, and CSF dynamics (Fultz et al., 2019; Helakari et al., 2022; Picchioni et al., 2022). Therefore, GS-related tissue and fluid changes may vary not only between wakefulness and sleep but also with sleep depth. Previous studies have demonstrated macroscopic CSF oscillations during NREM sleep and regional diffusion changes during sleep (Demiral et al., 2019; Fultz et al., 2019; Örzsik et al., 2023). However, it remains unclear whether time-dependent diffusion changes are coordinated across distributed brain regions during sleep.

Repeated multishell dMRI during sleep provides an opportunity to investigate this unresolved issue. Temporal changes in diffusion parameters can reflect changes in tissue water mobility during sleep. These changes may be influenced by ECS properties, vascular dynamics, and perivascular fluid movement, although dMRI provides indirect rather than direct evidence of these processes (Attwell et al., 2010; Jensen et al., 2005; Demiral et al., 2019; Örzsik et al., 2023; Sriram et al., 2024). Repeated measurements make it possible to examine not only regional diffusion changes but also whether these changes are coordinated across distributed brain regions. This approach is aligned with the GS as a brain-wide fluid transport process involving CSF influx, interstitial exchange, perivascular movement, and downstream drainage (Iliff et al., 2012; Xie et al., 2013).

In the present study, we combined simultaneous EEG with repeated multishell diffusion MRI acquired during in-scanner sleep to investigate sleep-depth-associated regional diffusion changes and their interregional organization. Scan-wise sleep depth corresponding to each diffusion MRI acquisition was quantified using the odds ratio product (ORP) (Younes et al., 2015), and diffusion tensor and diffusion kurtosis metrics were extracted from white-matter regions defined by the JHU ICBM-DTI-81 White-Matter Labels Atlas and gray-matter regions defined by the Automated Anatomical Labeling atlas, v. 2 (AAL2). We further evaluated the interregional organization of sleep-related diffusion changes using correlation network analysis.

## 2 Materials and methods

### 2.1 Participants and ethical approval

This study represents a secondary analysis of a previously reported sleep MRI dataset (Yun et al., 2025). Although based on the same experimental dataset, the present study addressed a distinct research question by investigating sleep-depth-associated regional diffusion tensor and diffusion kurtosis metrics and their interregional organization.

All experimental procedures were approved by the Institutional Review Board of Yonsei University MIRAE Campus (1041849-202305-BM-089-10), and written informed consent was obtained from all participants before the study. Twenty healthy male adults in their 20s were initially recruited. Individuals with a history of neurological or psychiatric disorders, night-shift work, or smoking more than five cigarettes per day were excluded. At enrollment, all participants had Pittsburgh Sleep Quality Index (PSQI) scores below 5 (Buysse et al., 1989), reported regular sleep habits, and had an estimated sleep efficiency of at least 90%. To facilitate stable sleep during the MRI, participants underwent 24 h of supervised sleep deprivation before the experiment and orally received 10 mg of physician-prescribed zolpidem 30 min before MRI acquisition. Compliance with the sleep-deprivation protocol was monitored every 30 min by two investigators through telephone calls and text messages. Throughout the sleep MRI session, EEG was continuously recorded to monitor the sleep state. Eight participants withdrew from the study before the completion of the imaging protocol because of newly reported claustrophobia or voluntary termination of the examination. Twelve participants completed the MRI protocol (mean age, 24.44 ± 2.50 years). Because the present study aimed to characterize diffusion changes across different sleep depths during continuous sleep, only participants who maintained uninterrupted sleep for at least 1 h during repeated diffusion MRI acquisitions were included in the final analysis. Consequently, data from nine participants were analyzed (mean age, 24.56 ± 2.74 years). Each participant underwent one diffusion MRI acquisition during wakefulness, followed by repeated diffusion MRI acquisitions during sleep. Between consecutive diffusion MRI acquisitions, 90-s diffusion gradient-free intervals were provided for EEG acquisition without gradient-related artifacts. The overall experimental timeline is summarized in Figure 1.

**Figure 1:**
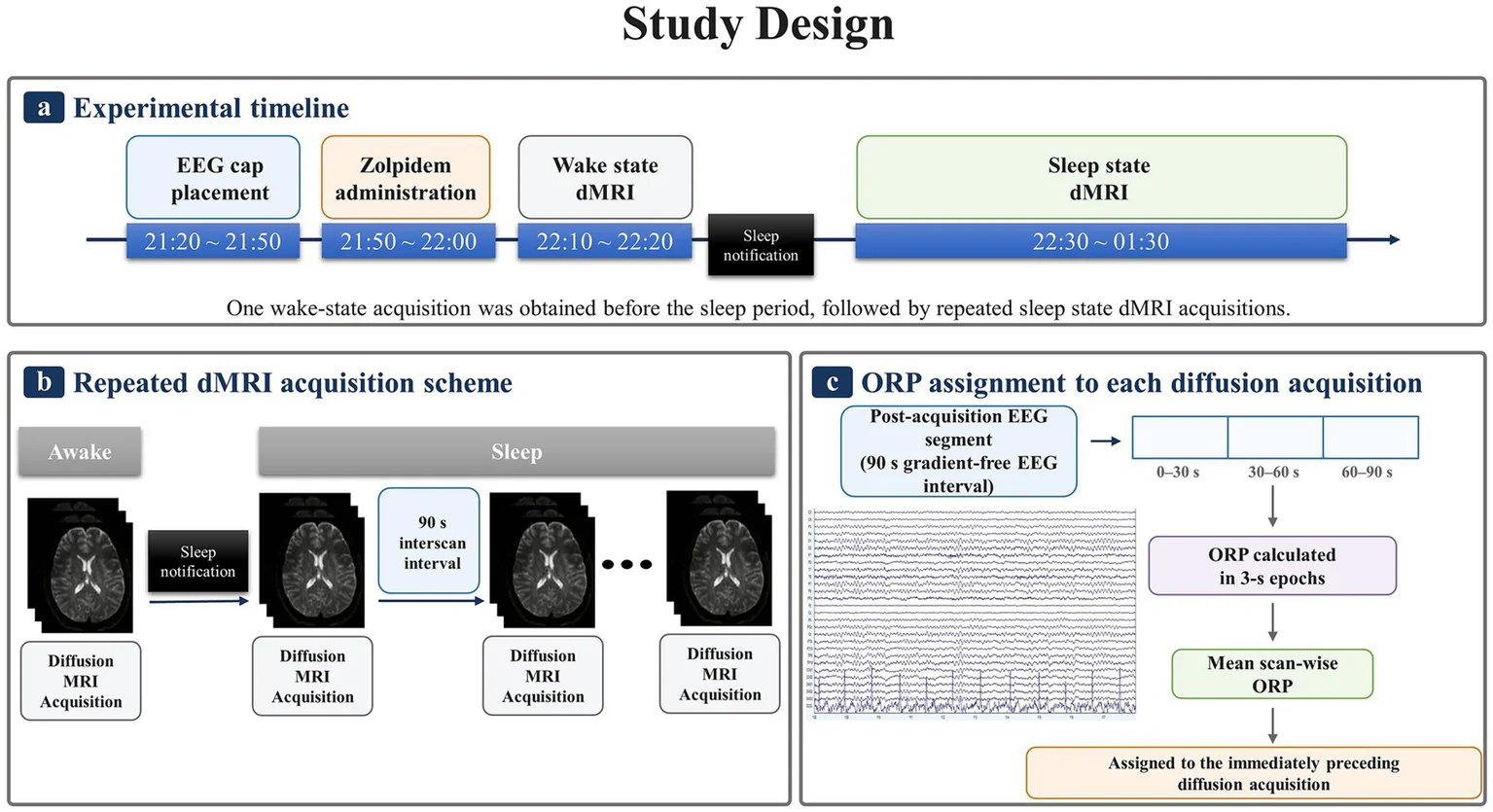
**(a)** Experimental timeline of the in-scanner sleep MRI protocol. After EEG cap placement, the participants received 10 mg zolpidem at approximately 22:00. One wake-state diffusion MRI acquisition was performed before the sleep period, followed by repeated sleep-state diffusion MRI acquisitions from approximately 22:30 to 01:30. **(b)** Each diffusion MRI acquisition was followed by a 90-s interscan interval. **(c)** ORP was calculated from consecutive 3-s epochs within each post-acquisition EEG segment, summarized across three consecutive 30-s intervals, and assigned to the immediately preceding diffusion acquisition as the scan-wise sleep-depth estimate.

### 2.2 Magnetic resonance imaging data acquisition

All MRI data were acquired using a 3-T MAGNETOM Skyra scanner (Siemens Healthcare, Erlangen, Germany) equipped with a 32-channel head coil. The diffusion imaging protocol followed the acquisition scheme established in a previous study (Yun et al., 2025), ensuring identical imaging conditions across participants. dMRI data were obtained using a multislice, single-shot diffusion-weighted echo-planar imaging sequence, with eight b0 (*b* = 0 s/mm^2^) images and two non-zero *b*-value shells (*b* = 800 and 2800 s/mm^2^), each sampled along 30 gradient directions. The imaging parameters were as follows: field of view (FOV) = 224 × 224 mm^2^, matrix size = 112 × 112, repetition time (TR) = 3800 ms, echo time (TE) = 105 ms, multiband factor = 3, slices = 78, voxel size = 1 × 1 × 2 mm^3^, and slice thickness = 2 mm with no slice gap. Data were acquired using anterior-to-posterior phase encoding, and eight b0 images were acquired using posterior-to-anterior phase encoding to estimate susceptibility-induced off-resonance fields for correction. A high-resolution structural T1-weighted image was also acquired for anatomical registration using a magnetization-prepared rapid gradient echo sequence (TR = 2300 ms, TE = 2.32 ms, flip angle = 9°, FOV = 256 × 256 mm^2^, isotropic voxel size = 0.9 mm). All axial images were acquired parallel to the anterior commissure–posterior commissure (AC–PC) line, as defined in the midsagittal plane. Each diffusion acquisition was followed by a 90-s gradient-free EEG interval, which was included to minimize MRI gradient-related EEG artifacts and to obtain an artifact-reduced sleep-depth estimate temporally adjacent to the preceding diffusion scan.

### 2.3 Magnetic resonance imaging data preprocessing

Diffusion preprocessing was performed using MRtrix3 (v. 3.0.3), FSL (v. 6.0.1), and DIPY (v. 1.8.0). Noise removal and Gibbs ringing correction were performed using MRtrix3. Thermal noise was reduced using Marchenko–Pastur principal component analysis denoising (Veraart et al., 2016), followed by Gibbs artifact suppression using the subvoxel-shift method (Kellner et al., 2016). Susceptibility-induced distortions were corrected with TOPUP (Andersson et al., 2003) using reversed-phase–encoding b0 images. Eddy-current distortions and head motion were corrected using eddy (Andersson and Sotiropoulos, 2016), with slice-to-volume motion correction, dynamic susceptibility correction, outlier replacement (−-*repol*), and b-vector rotation enabled. Brain extraction was performed using Brain Extraction Tool (BET) (Smith, 2002), and intensity inhomogeneity was corrected using N4 bias-field correction implemented in Advanced Normalization Tools (ANTs) (Tustison et al., 2010). To ensure consistent spatial resolution across the analyses, the preprocessed diffusion data were smoothed using a Gaussian kernel (full width at half maximum [FWHM] = 2.5 mm) and resampled to 2 × 2 × 2 mm^3^. Diffusion tensor and kurtosis parameters were estimated using DIPY (Garyfallidis et al., 2014; Tabesh et al., 2011), with a weighted linear least-squares approach and positivity constraints yielding eight parameter maps: fractional anisotropy (FA), mean diffusivity (MD), axial diffusivity (AD), radial diffusivity (RD), mean kurtosis (MK), axial kurtosis (AK), radial kurtosis (RK), and kurtosis fractional anisotropy (KFA).

### 2.4 Spatial normalization and region-of-interest time-series extraction

All diffusion parameter maps were spatially normalized to the MNI152 template space using individual T1-weighted images. Because the diffusion parameter maps were initially defined in the diffusion space, the individual b0 images were first rigidly coregistered to each participant’s T1-weighted image. Subsequently, the resulting b0-to-T1 transformation was applied to the diffusion parameter maps. Each participant’s T1-weighted image was nonlinearly normalized to the MNI152 template using SPM12 (Friston et al., 1994). The resulting deformation field was applied to the diffusion parameter maps that were already aligned to the T1 space. Atlas-based regions of interest (ROIs) were defined separately for WM and GM. WM ROIs were defined using the JHU ICBM-DTI-81 atlas (Mori et al., 2008), and GM ROIs were defined using the automated anatomical labeling atlas, v. 2 (AAL2) (Tzourio-Mazoyer et al., 2002). All atlas masks were resampled, thresholded, and binarized in a normalized diffusion space to match the grid of the Montreal Neurological Institute (MNI)-transformed diffusion parameter maps. Because EEG gel and electrode placement predominantly affected signal quality in the right hemisphere, all analyses were restricted to the left hemisphere for both WM and GM. For each diffusion parameter and time point, the mean value within each ROI was extracted, yielding parameter-specific ROI time series for each participant (comprising 28 WM and 64 GM ROIs). Outlier volumes exceeding three standard deviations from a participant’s ROI mean were replaced using linear interpolation.

### 2.5 Electroencephalography preprocessing

EEG was recorded throughout the dMRI session using a 32-channel BrainAmp system (Brain Products GmbH, Germany) at 5,000 Hz, with a hardware band-pass filter of 0.1–250 Hz. Electrode impedances were maintained below 5 kΩ. For EEG analysis, only the 90-s post-acquisition EEG recordings acquired between consecutive diffusion MRI acquisitions were used. These recordings were obtained during periods without MRI data acquisition, thereby avoiding MRI acquisition-related gradient artifacts. These intervals were down-sampled to 512 Hz, band-pass filtered between 1 and 35 Hz, and subjected to independent component analysis (ICA) to remove pulse artifacts (Srivastava et al., 2005). The ICA classifications were independently performed by two trained researchers, and only components identified by both raters as pulse-artifact-related were removed. Preprocessing was performed using EEGLAB (v. 2023.0) and custom MATLAB routines. The final artifact-cleaned EEG recording for each segment (~90 s) was reviewed by three board-certified EEG specialists to confirm physiologically interpretable wake/sleep states and exclude residual non-physiological activity. Because the recorded channels did not include full polysomnography (e.g., electromyography and electrooculography) and EEG was acquired intermittently between diffusion scans, stage-specific scoring (N1/N2/N3) was not performed; instead, experts provided coarse wake-versus-sleep classifications.

### 2.6 Odds ratio product (ORP) quantification

ORP was computed as described by Younes et al. (2015), quantifying sleep depth as an arousal-propensity index based on rank-based spectral power in the delta (0.33–2.33 Hz), theta (2.67–6.33 Hz), alpha (7.33–14 Hz), and beta (14.33–35 Hz) frequency bands. Because the original ORP lookup tables are not publicly available, we reconstructed an ORP reference model using a dataset from 5,792 participants in the Sleep Heart Health Study (SHHS) (Quan et al., 1997; Zhang et al., 2018). The dataset was randomly split into training (80%) and testing (20%) sets. Spectral deciles were computed for each frequency band from the training set and used to generate rank-based ORP assignment tables. The tables were then applied to the test set to confirm the stage-ordered ORP distributions and wake-versus-non-wake discrimination. After validation, the SHHS-derived ORP table was applied to the EEG recordings using central channels C3 and C4. The ORP was computed separately for C3 and C4 and averaged when both channels were available.

ORP was calculated from consecutive 3-s epochs within each 90-s post-acquisition EEG segment. For scan-level analyses, each post-acquisition EEG segment was divided into three consecutive 30-s intervals. Within each interval, the ORP was summarized as the mean across valid 3-s epochs. The scan-level ORP assigned to each diffusion MRI acquisition was defined as the mean of the three 30-s interval means derived from the corresponding post-acquisition EEG segment, yielding one ORP value per diffusion MRI acquisition for subsequent analyses.

To further characterize the stability of scan-wise ORP estimates, we quantified within-segment ORP variability across the 72 post-acquisition EEG segments corresponding to the eight sleep-state diffusion MRI acquisitions in nine participants. ORP variability was summarized using the standard deviation (SD) and range of consecutive 3-s ORP values, as well as the SD and range of the three 30-s interval means. Conventional relative delta power was calculated from the same EEG segments as power in the 0.5–4 Hz frequency band normalized to the total power in the 0.5–35 Hz frequency band. Associations between scan-wise ORP and relative delta power were evaluated using both pooled and subject-wise Spearman’s correlation analyses. Subject-wise correlation coefficients were subsequently tested against zero using the Wilcoxon signed-rank test.

### 2.7 Odds ratio product diffusion association analysis and paired tensor–kurtosis consistency

To assess the relationship between the ORP values and diffusion parameters, Spearman’s rank correlation coefficients (*ρ*) were calculated for each participant, ROI, and diffusion parameter using repeated acquisitions. Group-level effects were summarized as the median ρ across participants and tested against zero using two-sided one-sample Wilcoxon signed-rank tests. ROIs were screened for significant ORP–diffusion associations using a dual criterion. First, an effect-size threshold of |median *ρ*| ≥ 0.25 was applied to retain effects of at least small-to-moderate magnitude (Schönbrodt and Perugini, 2013). Second, participant-wise correlation coefficients were required to yield a group-level Wilcoxon *p* < 0.05. Multiple-comparison correction was not applied at this screening stage because the purpose was to identify candidate ROIs for subsequent robustness filtering based on paired tensor–kurtosis consistency and network-level recurrence. ROIs were then evaluated across predefined tensor–kurtosis pairs (FA–KFA, AD–AK, MD–MK, and RD–RK) and retained when both parameters within a pair showed significant ORP–diffusion associations.

### 2.8 Correlation network construction and hub definition

Parameter-specific correlation networks were constructed separately for WM and GM. For each participant and diffusion parameter, an ROI-by-ROI Spearman’s correlation matrix was computed by correlating the ROI time series across repeated acquisitions. Correlation coefficients were Fisher z-transformed to stabilize variance and reduce bias during averaging. These were then averaged across participants and inverse transformed back to the correlation values for reporting (Fisher, 1915). To obtain weighted adjacency matrices for graphical analysis, we restricted networks to positive correlations (*ρ* > 0) and used proportional thresholding to harmonize edge densities across parameters. Network density was scanned from 10 to 30% in 1% increments. For each density, modularity (Q) was computed using the Louvain community detection algorithm (Blondel et al., 2008). For each parameter, the final density was selected to yield a stable modular organization across adjacent densities and a modularity value of approximately 0.30 (Q ≈ 0.30), a commonly used lower-bound criterion for non-random community structure in empirical networks (Clauset et al., 2004). This Q-based rule was used as an operational criterion to avoid extremely sparse or overly dense graphs and to obtain an interpretable network structure; it was not treated as a biological target. Nodal strength was computed as the sum of the weights of all positive edges connected to each node. Threshold selection and the resulting modularity–density curves for all diffusion tensor and kurtosis parameters are shown in Supplementary Figure S1, which shows the behavior of modularity across density thresholds for the WM and GM networks. In addition to nodal strength, we computed the nodal clustering coefficient and local efficiency as well as the global metrics of global efficiency and characteristic path length, using established weighted-network definitions and interpretation guidelines (Rubinov and Sporns, 2010; Masuda et al., 2025). To quantify small-world organization, we generated degree-preserving randomized surrogate networks (1,000 iterations per parameter network) and expressed the clustering coefficients and characteristic path lengths as ratios relative to the surrogate means (*γ* and *λ*, respectively). Small-worldness (*σ*) was computed from these ratios, and global efficiency was reported as a ratio relative to the surrogate ensemble. Hubs were defined as nodes with nodal strength values exceeding z > 1.28 relative to the within-network distribution, corresponding to the upper decile under a normal approximation. We identified hub regions that also showed significant ORP–diffusion associations (hereafter termed sleep-associated hubs [SAHs]).

### 2.9 Global covariance control, key-edge identification, and odds ratio product–dependent typology

To attenuate the global shared covariance that can inflate pairwise correlations, we controlled for the first principal component (PC1), which represents the largest component of variance shared across ROIs. Because PC1 may reflect contributions from sleep-depth-related physiological variations, scan progression, vascular or pulsatile effects, and other common sources of covariance, it was not interpreted as representing noise alone. Instead, adjustment for PC1 was used as a conservative control for global covariance before evaluating sleep-depth-associated interregional correlations. For each diffusion parameter, ROI time series were z-scored separately for each participant and concatenated across participants to form a pooled sample-by-ROI matrix. Principal component analysis was applied to this pooled matrix, and the PC1 score time course was regressed from each ROI time series to obtain the PC1-controlled ROI time courses (residual signals). ROI-to-ROI Spearman correlations were then recomputed using the PC1-controlled time courses and thresholded using the Benjamini–Hochberg false discovery rate (BH-FDR) correction at *q* < 0.05, retaining only positive edges (*ρ* > 0). For multiparameter key-edge recurrence, we focused on three matched tensor–kurtosis pairs (AD–AK, RD–RK, and MD–MK), which yielded six parameter networks (AD, RD, MD, AK, RK, and MK). We did not include FA and KFA in this intersection, as they are composite anisotropy indices derived from multiple eigenvalue terms and are less directly interpretable as directional tensor–kurtosis counterparts in a key-edge framework. Key edges were defined as ROI pairs that survived the BH-FDR correction in all six PC1-controlled parameter networks. These represented high-confidence multiparameter interregional correlations. To test sleep depth sensitivity, ORP was additionally regressed from each PC1-controlled ROI time course (ordinary least squares), yielding ORP-controlled time courses. Parameter-specific networks were recomputed on ORP-controlled time courses and thresholded using BH-FDR *q* < 0.05. Each key edge was classified as ORP-independent if it remained significant across all six ORP-controlled networks, or ORP-dependent otherwise, indicating sensitivity to sleep depth. PC1 control was applied to attenuate broad shared variance that can inflate correlations, whereas subsequent ORP control was used to identify interregional correlations sensitive to the scan-wise sleep-depth estimate.

### 2.10 Vascular-territory assignment

Vascular-territory labels used in the key-edge analyses were assigned in the MNI space using the MNI vascular-territory atlas (Schirmer et al., 2019). This atlas provides supratentorial vascular territories for the anterior cerebral artery (ACA), middle cerebral artery (MCA), and posterior cerebral artery (PCA). For each supratentorial GM ROI, voxel-wise overlap with the vascular territory map was computed, and the ROI was assigned to the territory showing the largest overlap among the ACA, MCA, and PCA. Because infratentorial vascular territories were not separately defined in this atlas, cerebellar and vermian ROIs were classified as vertebrobasilar artery (VBA)/posterior circulation regions for descriptive key-edge analysis. These vascular labels were used to classify key edges as same-territory (ST) or cross-territory (CT) connections across the ACA, MCA, PCA, and VBA categories. To account for anatomical base rates, we also quantified the number of ROIs and possible ROI-to-ROI pairs within each vascular-territory category and compared the observed distribution of sleep-depth-sensitive key edges with the distribution expected from the number of possible ROI pairs.

### 2.11 Statistical analysis

All analyses were performed using MATLAB R2023a and Python software. Spearman’s rank correlation was used to quantify monotonic associations between ORP and diffusion parameters. Group-level ORP–diffusion effects were assessed using a two-sided one-sample Wilcoxon signed-rank test applied to participant-wise correlation coefficients. BH-FDR correction was applied at *q* < 0.05 across all ROI pairs, retaining only positive edges. Graph-theoretical metrics, including nodal strength, clustering coefficient, local efficiency, characteristic path length, and global efficiency, were computed using standard weighted-network definitions. For small-world normalization, we generated degree-preserving randomized surrogate networks (1,000 iterations per parameter network) and computed the normalized clustering coefficient and characteristic path length relative to these surrogate networks.

Additional robustness and sensitivity analyses were performed in response to the exploratory nature of the ROI and network analyses. First, ROI-screening flow counts were summarized by reporting the number of ROI–parameter tests retained at the nominal association stage, after FDR correction, and after paired tensor–kurtosis consistency filtering. Second, leave-one-subject-out (LOSO) analyses were performed by excluding one participant at a time and repeating the group-level ROI–ORP association tests. Third, PC1 was characterized by reporting the percentage of variance explained and its Spearman’s correlation with ORP. Fourth, network-level sensitivity was assessed by comparing edge recurrence in networks with and without PC1 regression and by repeating recurrent-edge analysis across fixed absolute-correlation thresholds. Finally, a vascular-territory base-rate analysis was performed to determine whether the observed distribution of sleep-depth-sensitive edges exceeded the distribution expected from the number of possible ROI pairs within each vascular category.

## 3 Results

### 3.1 Odds ratio product validation and scan-wise EEG characterization

To validate the SHHS-derived ORP reconstruction, we compared the reconstructed ORP values with scorer-annotated sleep stages in the SHHS test dataset. The cumulative distribution functions showed monotonic separation across stages, with the highest ORP during wakefulness and progressively lower values from N1 to N3 (Figure 2a). Stage-wise notched boxplots further showed distinct median ORP values across the wake, N2, and N3 stages (Figure 2b). In a representative overnight recording, transitions among the wakefulness, NREM, and rapid eye movement (REM) stages were tracked using 30-s ORP values. These transitions decreased during sustained NREM and showed transient increases during brief arousals or REM (Figure 2c). A receiver operating characteristic analysis for classifying wake versus non-wake epochs yielded an area under the curve of 0.931 (Figure 2d). This indicated a strong correspondence between the reconstructed ORP values and arousal states. These results support the use of reconstructed ORP as a scan-wise sleep depth for subsequent ROI-level ORP–diffusion association and network analyses.

**Figure 2:**
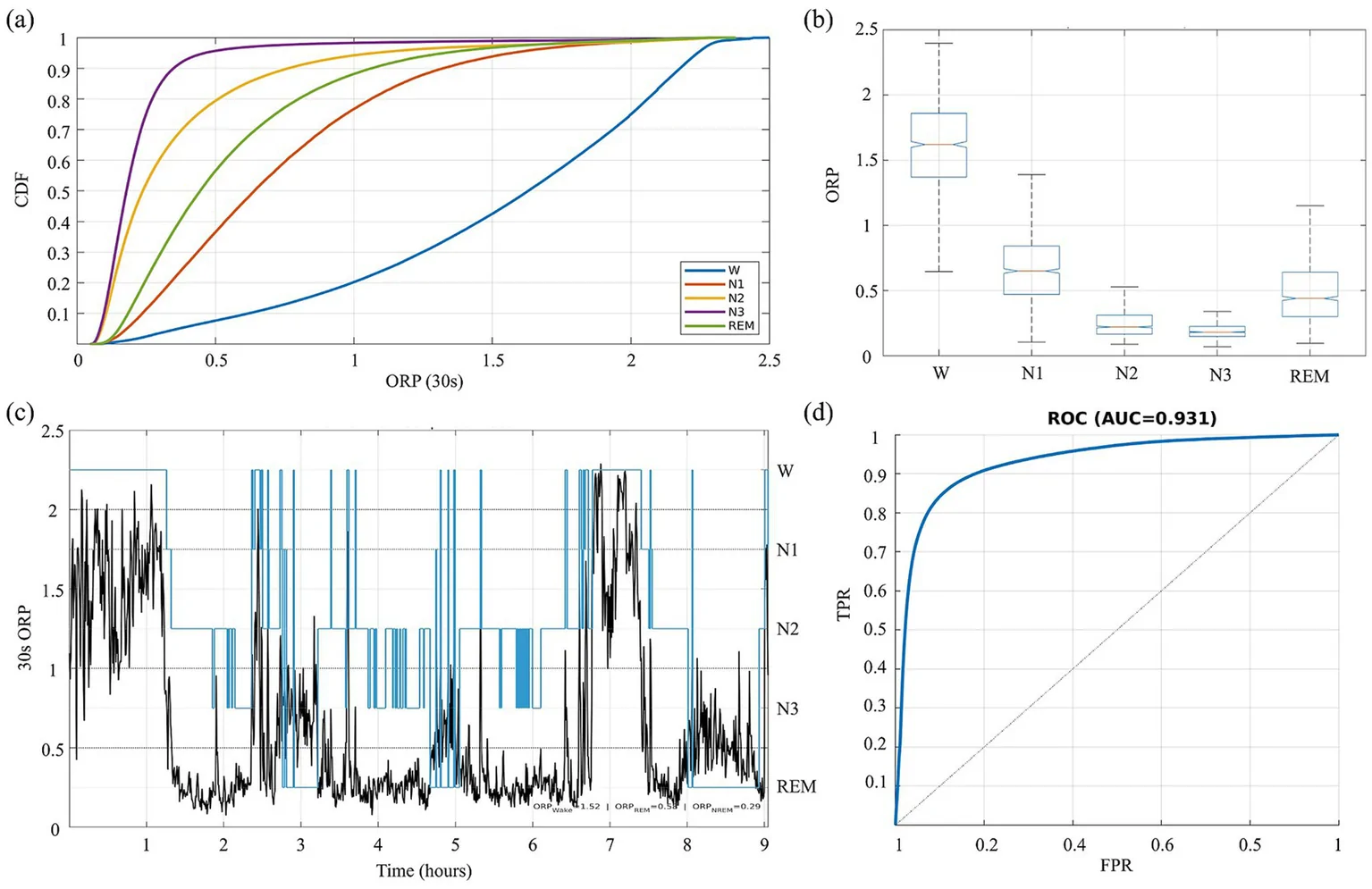
Validation of the reconstructed odds ratio product (ORP). **(a)** Cumulative distribution functions (CDFs) of 30-s ORP values stratified by scorer-defined sleep stage: Wakefulness (W), N1, N2, N3, and rapid eye movement (REM) sleep. **(b)** Notched boxplots showing the distribution of ORP values across sleep stages. **(c)** Representative overnight recording showing the temporal correspondence between 30-s ORP values and scorer-defined sleep stages. **(d)** Receiver operating characteristic (ROC) curve for ORP-based classification of wake versus non-wake epochs (AUC = 0.931).

To further characterize the scan-wise sleep-depth estimates in the present MRI dataset, we quantified the ORP variability within the 72 post-acquisition EEG segments corresponding to the eight sleep-state diffusion MRI acquisitions in nine participants. The median segment duration was 85.85 s (interquartile range [IQR], 83.00–89.00 s). The ORP calculated from consecutive 3-s epochs showed within-segment variability, with a median 3-s ORP SD of 0.233 (IQR, 0.138–0.371) and a median 3-s ORP range of 1.051 (IQR, 0.589–1.419). When ORP was summarized across three consecutive 30-s intervals, variability was substantially smaller, with a median 30-s ORP SD of 0.070 (IQR, 0.032–0.143) and a median 30-s ORP range of 0.129 (IQR, 0.056–0.272; Supplementary Table 10).

We also compared scan-wise ORP with conventional relative delta power calculated from the same EEG segments. ORP showed the expected inverse association with relative delta power, with a pooled Spearman’s correlation of *ρ* = −0.515 across the 72 segments and a subject-wise median correlation of ρ = −0.333 (IQR, −0.810 to −0.214). Subject-wise correlations were significantly below zero (Wilcoxon signed-rank, *p* = 0.0078). These findings provide an internal EEG-based check that the scan-wise ORP estimates captured the expected sleep-depth-related spectral variation, while indicating that short-epoch ORP fluctuations were attenuated by 30-s interval averaging.

### 3.2 Odds ratio product diffusion associations and regions of interest retained across matched tensor–kurtosis pairs

Because a lower ORP indicates deeper sleep, a positive ORP–diffusion parameter correlation indicates that the diffusion parameter decreases as sleep deepens. Conversely, a negative correlation indicates an increased diffusion parameter with deeper sleep. Supplementary Table S1 lists all WM and GM ROIs with significant ORP–diffusion associations. To prioritize the effects supported by both tensor and kurtosis parameters, we evaluated predefined tensor–kurtosis pairs (FA–KFA, AD–AK, MD–MK, and RD–RK) and retained only those ROIs in which both parameters within a pair showed significant ORP–diffusion associations. Table 1 shows the retained ROIs for the matched tensor–kurtosis pairs. In WM, paired FA–KFA associations were observed in the left corticospinal tract and sagittal stratum, with FA, a diffusion anisotropy index, and KFA, a kurtosis anisotropy index, both of which were positively correlated with ORP. Because lower ORP indicates deeper sleep, these findings indicate that deeper sleep was associated with reductions in both diffusion anisotropy and kurtosis anisotropy in these WM pathways. Diffusivity–kurtosis opposition was observed in the cerebellum (Vermis_3). MD and RD decreased with deeper sleep, whereas MK and RK increased. In the GM, paired tensor–kurtosis effects were most prominent in the sensorimotor cortex (Precentral_L, Postcentral_L, supplementary motor area, and paracentral lobule) and extended to the frontal and parietal association cortices. Across these GM regions, deeper sleep was generally associated with decreased diffusivity (AD/MD/RD) and increased kurtosis (AK/MK/RK), indicating coordinated modulation of tensor and kurtosis parameters within the same ROIs.

**Table 1:** ROIs retained across matched tensor–kurtosis pairs in the analysis of sleep-depth-associated diffusion parameter changes.

Cluster	ROI	Diffusion parameters (pair type)	ORP–diffusion association (tensor, kurtosis)	*p*-value
White matter tracts	Corticospinal tract L	(FA, KFA)	(0.3333, 0.3000)	(0.0195, 0.0078)
	Sagittal stratum L	(FA, KFA)	(0.4333, 0.4167)	(0.0195, 0.0195)
Cerebellum	Vermis_3	(MD, MK)	(0.4167, −0.3667)	(0.0078, 0.0039)
		(RD, RK)	(0.4500, −0.3167)	(0.0078, 0.0117)
Basal ganglia	Putamen_L	(FA, KFA)	(0.3167, 0.2500)	(0.0391, 0.0499)
Sensorimotor cortex	Precentral_L	(AD, AK)	(0.4833, −0.2500)	(0.0039, 0.0078)
		(MD, MK)	(0.4667, −0.3000)	(0.0039, 0.0116)
		(RD, RK)	(0.5000, −0.3500)	(0.0039, 0.0078)
	Postcentral_L	(AD, AK)	(0.3667, −0.3000)	(0.0039, 0.0195)
		(MD, MK)	(0.3833, −0.3000)	(0.0117, 0.0117)
	Supp_Motor_Area_L	(AD, AK)	(0.4167, −0.3167)	(0.0039, 0.0039)
	Paracentral_Lobule_L	(AD, AK)	(0.4000, −0.2833)	(0.0039, 0.0039)
		(MD, MK)	(0.4000, −0.2667)	(0.0117, 0.0078)
Cingulate–prefrontal executive regions	Frontal_Mid_2_L	(MD, MK)	(0.5000, −0.3667)	(0.0039, 0.0078)
		(RD, RK)	(0.4333, −0.3833)	(0.0039, 0.0039)
	Frontal_Sup_2_L	(AD, AK)	(0.5500, −0.3667)	(0.0039, 0.0078)
		(MD, MK)	(0.5500, −0.3833)	(0.0039, 0.0039)
		(RD, RK)	(0.5500, −0.4500)	(0.0039, 0.0039)
	OFClat_L	(FA, KFA)	(0.4268, 0.3667)	(0.0195, 0.0039)
Association cortices	Parietal_Inf_L	(RD, RK)	(0.3667, −0.3000)	(0.0391, 0.0039)
Temporal cortex	Temporal_Sup_L	(RD, RK)	(0.4686, −0.3500)	(0.0078, 0.0273)

ROIs were included only if both parameters within a predefined tensor–kurtosis pair (FA–KFA, AD–AK, MD–MK, and RD–RK) showed significant ORP–diffusion associations (|median *ρ*| ≥ 0.25 and group-level Wilcoxon *p* < 0.05). The values shown represent the median Spearman correlation coefficients across participants.

### 3.3 Parameter-specific region-of-interest correlation matrices

Figure 3 shows the group-level ROI-to-ROI Spearman’s correlation matrices for the representative diffusion parameters (FA and KFA in WM; RD and RK in GM). These parameters were selected to capture the dominant diffusion patterns in each tissue type. The matrices for all diffusion parameters are provided in Supplementary Figure S2. For each parameter, the participant-level correlation matrices were Fisher z-transformed, averaged across participants, and inverse transformed back to correlation values. Each matrix element reflects the degree to which two regions exhibited coordinated changes in their diffusion parameters across repeated diffusion acquisitions during sleep. These matrices provided the basis for subsequent graph-theoretical analyses. Visualizations of the full left-hemisphere and midline ROI networks for all parameters are provided in Supplementary Figure S3.

**Figure 3:**
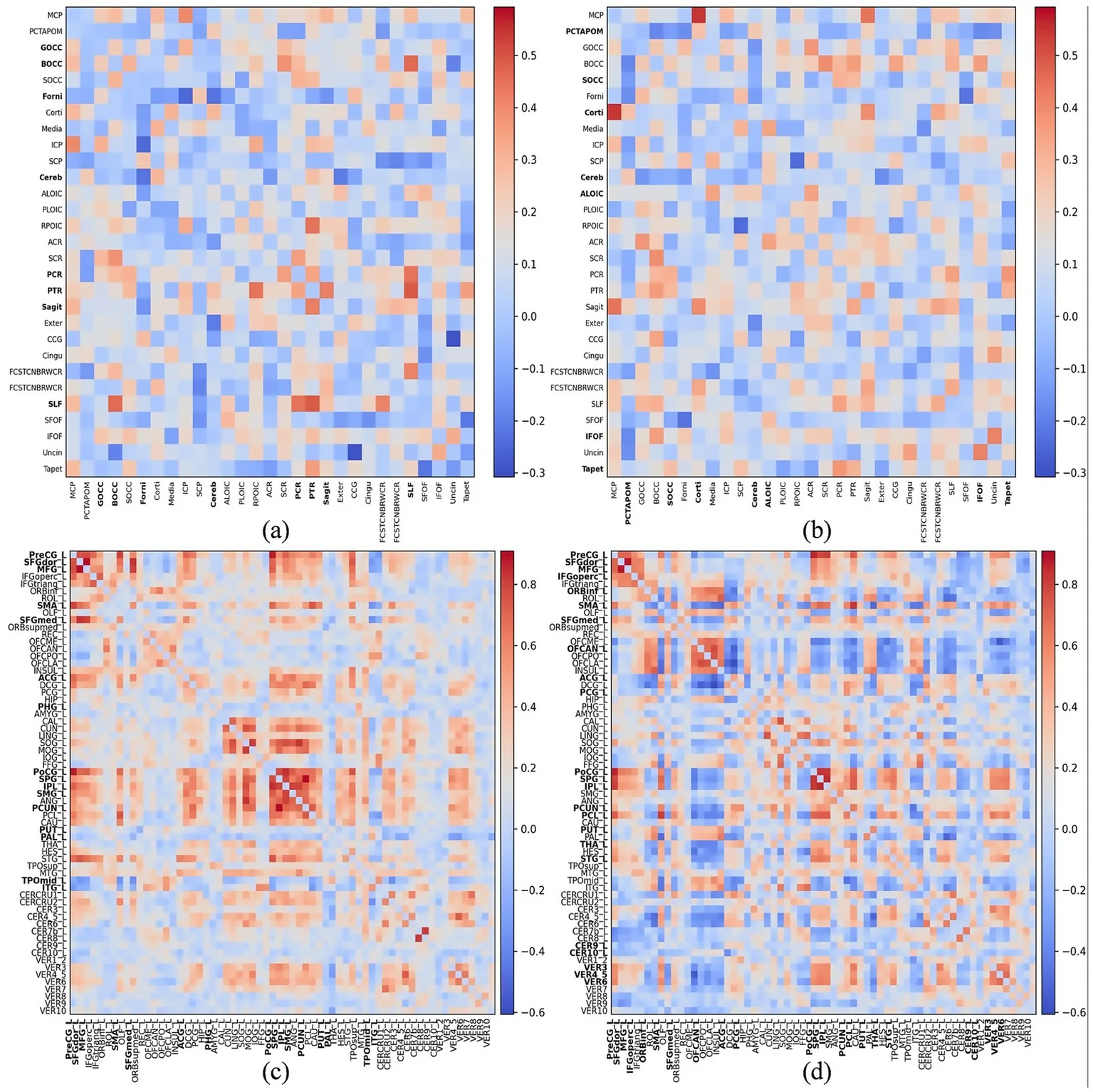
Correlation matrices illustrating interregional correlations in representative diffusion parameters. **(a,b)** WM: FA and KFA show structured interregional correlations associated with anisotropy-related diffusion changes. **(c,d)** GM: RD and RK show consistent diffusion–kurtosis relationships across regions. These parameters were selected to represent the dominant diffusion patterns observed in each tissue type. Full correlation matrices for all diffusion parameters are provided in Supplementary Figure S2.

### 3.4 Identification of sleep-associated hub nodes

Nodes with nodal strength z-scores > 1.28 in the positive-weight networks were defined as hub nodes. These were intersected with ROIs showing significant ORP–diffusion associations. The resulting SAH nodes represent ROIs that were both topologically central and associated with sleep depth. In the GM, the SAH nodes were concentrated in the left sensorimotor and parietal territories. Precentral and postcentral regions appeared repeatedly across AD, AK, MD, MK, RD, and RK. Additional SAH nodes were observed in the supplementary motor area, paracentral lobule, inferior and superior parietal regions, supramarginal gyrus, and superior temporal cortex. KFA identified a single GM SAH node in the inferior temporal gyrus. In WM, SAH nodes were fewer and confined to major projection and association pathways. These included the posterior thalamic radiation and inferior fronto-occipital fasciculus for FA and the sagittal stratum for KFA. Overall, SAH overlap was consistently concentrated in sensorimotor–parietal GM territories, whereas WM overlap was sparse and pathway-specific.

### 3.5 Graph-theoretical topology of region-of-interest correlation networks

The network topology metrics for the diffusion tensor and kurtosis correlation networks are presented in Table 2. Across the parameters, the small-worldness index (*σ*) was generally greater than 1. Clustering ratios (*γ*) were consistently greater than 1 in both WM and GM. Path-length ratios (*λ*) were modestly greater than 1 relative to degree-matched random networks, whereas the global efficiency ratios were below, but close to, 1. Overall, these findings suggest a modest small-world-like organization, characterized by locally clustered networks that retained a non-random integrative structure across parameters.

**Table 2:** Network topology metrics across diffusion tensor and kurtosis parameters.

Tissue	Diffusion parameter	Small-worldness index (σ)	Clustering ratio (γ)	Path-length ratio (λ)	Global efficiency ratio
WM	AD	1.31 ± 0.32	1.56 ± 0.39	1.19 ± 0.03	0.91 ± 0.01
	AK	1.34 ± 0.34	1.57 ± 0.43	1.17 ± 0.04	0.92 ± 0.02
	FA	1.29 ± 0.26	1.54 ± 0.34	1.19 ± 0.09	0.91 ± 0.04
	KFA	1.35 ± 0.31	1.54 ± 0.35	1.14 ± 0.06	0.93 ± 0.03
	MD	1.27 ± 0.22	1.52 ± 0.29	1.19 ± 0.05	0.91 ± 0.02
	MK	1.24 ± 0.29	1.58 ± 0.45	1.27 ± 0.21	0.89 ± 0.05
	RD	1.34 ± 0.23	1.59 ± 0.32	1.18 ± 0.06	0.91 ± 0.03
	RK	1.25 ± 0.18	1.55 ± 0.29	1.24 ± 0.11	0.89 ± 0.04
GM	AD	1.20 ± 0.43	1.59 ± 0.56	1.33 ± 0.08	0.86 ± 0.03
	AK	1.28 ± 0.41	1.63 ± 0.48	1.28 ± 0.09	0.87 ± 0.03
	FA	1.30 ± 0.32	1.59 ± 0.38	1.23 ± 0.05	0.88 ± 0.02
	KFA	1.35 ± 0.37	1.64 ± 0.48	1.22 ± 0.09	0.89 ± 0.04
	MD	1.18 ± 0.37	1.55 ± 0.49	1.32 ± 0.11	0.86 ± 0.05
	MK	1.11 ± 0.16	1.48 ± 0.25	1.33 ± 0.07	0.85 ± 0.03
	RD	1.23 ± 0.41	1.62 ± 0.55	1.32 ± 0.12	0.86 ± 0.05
	RK	1.04 ± 0.13	1.43 ± 0.26	1.37 ± 0.11	0.83 ± 0.04

The values shown are presented as the mean ± standard deviation across participants for the σ, γ, λ, and global efficiency ratios.

### 3.6 Multiparameter key edges and odds ratio product dependence

To identify robust interregional correlations shared across parameter-specific networks, we performed multiparameter key-edge selection using PC1-controlled ROI time courses. We constructed ROI correlation networks for six parameters from three matched tensor–kurtosis pairs (AD/AK, RD/RK, and MD/MK). The positive edges that survived the BH-FDR correction (*q* < 0.05) within each parameter network were retained. Intersecting the six parameter-specific networks yielded 36 recurrent multiparameter key edges (Table 3). Of these, 25 connected ROIs were within the ST, and 11 were within the CT. To assess the contribution of sleep depth to parameter changes, we re-estimated the six parameter networks using ORP-controlled time courses and applied the same BH-FDR procedure. Key edges that remained significant in all six ORP-controlled networks were classified as ORP-independent, and those that did not were classified as ORP-dependent (Table 3). Overall, 29 edges were ORP-independent (19 ST, 10 CT) and 7 were ORP-dependent (6 ST, 1 CT).

**Table 3:** Multiparameter key edges by odds ratio product dependence and vascular territory.

ORP typology	Cross-vascular territory	Same vascular territory	Total
ORP-independent	10	19	29
ORP-dependent	1	6	7
Total	11	25	36

ROI pairs that survived the BH-FDR correction (*q* < 0.05) in all six parameter networks estimated from the PC1-controlled time courses (AD, RD, MD, AK, RK, and MK) were defined as key edges. After ORP control, the edges retained in all six networks were classified as ORP-independent, and those that were not (retained in fewer than six networks) as ORP-dependent.

The seven ORP-dependent key edges are listed in Table 4. They included MCA within-territory frontal–insular/opercular regions (Frontal_Inf_Oper_L–Insula_L; Insula_L–Rolandic_Oper_L), orbitofrontal (Frontal_Inf_Orb_2_L–OFCpost_L), and temporo-parietal (SupraMarginal_L–Temporal_Inf_L) pairs, and the posterior circulation/ VBA cerebellar and vermian pairs (Cerebelum_7b_L–Cerebelum_8_L; Vermis_7–Vermis_8). A single ORP-dependent CT edge linked the ACA and MCA sensorimotor regions (Paracentral_Lobule_L–Precentral_L). A full list of all 36 key edges and their vascular-territory labels is provided in Supplementary Table S2.

**Table 4:** ORP-dependent key edges classified as sleep-depth-sensitive candidate interregional correlations.

ROI 1	ROI 2	ST/CT	Territory 1	Territory 2	Endpoint category
Frontal_Inf_Oper_L	Insula_L	ST	MCA	MCA	Significant ORP–diffusion association
Insula_L	Rolandic_Oper_L	ST	MCA	MCA	Neither
SupraMarginal_L	Temporal_Inf_L	ST	MCA	MCA	SAH
Frontal_Inf_Orb_2_L	OFCpost_L	ST	MCA	MCA	Neither
Cerebelum_7b_L	Cerebelum_8_L	ST	VBA	VBA	Significant ORP–diffusion association
Vermis_7	Vermis_8	ST	VBA	VBA	Neither
Paracentral_Lobule_L	Precentral_L	CT	ACA	MCA	SAH

Each row lists an ORP-dependent key edge that did not remain significant across all six ORP-controlled parameter networks. The territories were labeled as anterior cerebral artery (ACA), middle cerebral artery (MCA), or posterior circulation, including the vertebrobasilar artery (VBA)/cerebellar regions. The endpoint category column indicates whether either endpoint showed a significant ORP–diffusion association and/or was an SAH node in the PC1-controlled networks.

Because the MCA territory contains a relatively large number of possible ROI pairs, the anatomical base rate of vascular-territory assignments was evaluated separately in the robustness and sensitivity analysis.

To visualize the anatomical distribution of these sleep-depth-sensitive candidate edges, the seven ORP-dependent key edges were mapped onto an AAL2-derived brain surface (Figure 4). The visualization demonstrated that the MCA edges were distributed across lateral frontal, insular/opercular, orbitofrontal, and temporoparietal cortical regions, whereas the VBA edges were localized to cerebellar and vermian regions, and the single CT edge involved the medial sensorimotor region.

**Figure 4:**
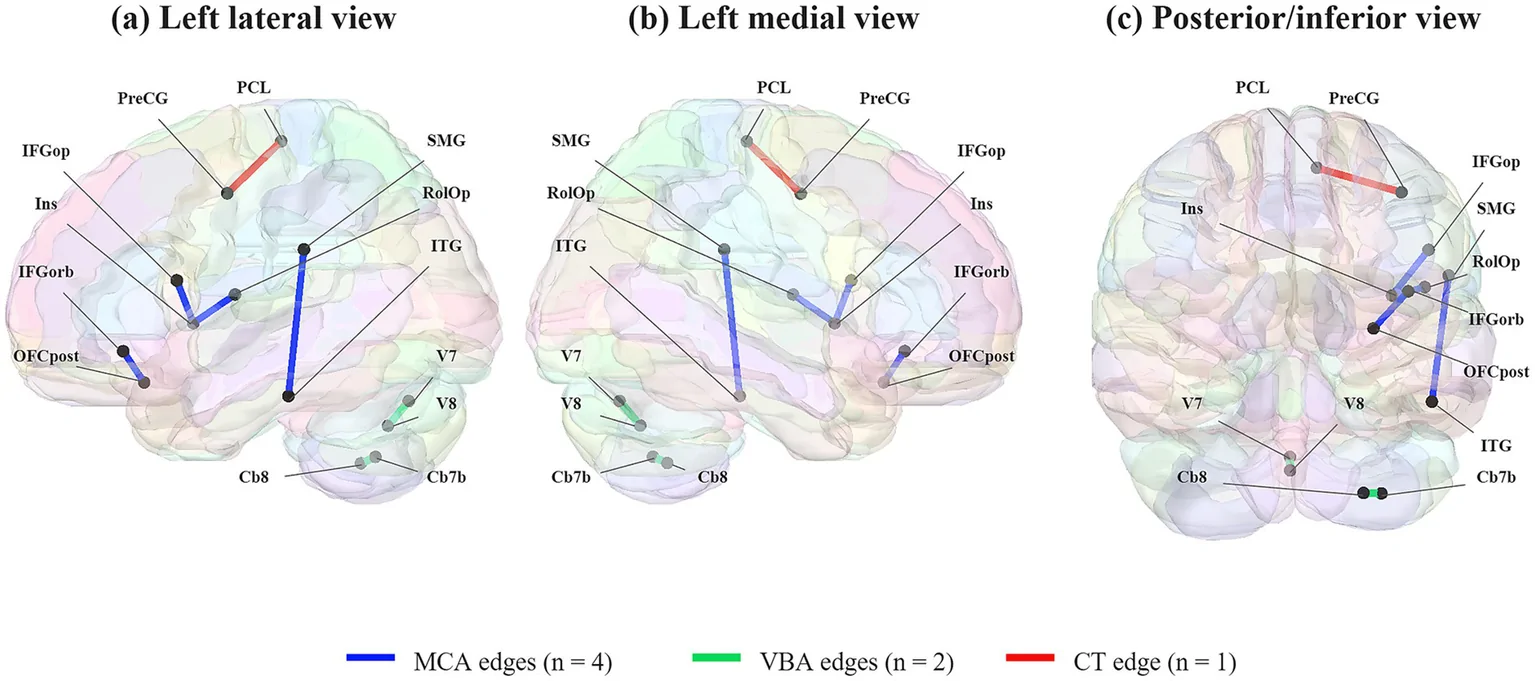
Three-view brain-space visualization of ORP-dependent key edges. **(a)** Left lateral, **(b)** Left medial, and **(c)** Posterior/inferior views of the seven ORP-dependent key edges classified as sleep-depth-sensitive candidate interregional correlations. Four edges were located within the middle cerebral artery (MCA) territory (blue), two were located within the posterior-circulation cerebellar and vermian regions (VBA, green), and one cross-territory edge connected the anterior and middle cerebral artery territories (CT, red). Black spheres indicate ROIs participating in these ORP-dependent edges. Edges represent the interregional temporal covariation of diffusion parameters across repeated sleep acquisitions and do not indicate anatomical connectivity, direct functional connectivity, directed interactions, or direct transport pathways.

### 3.7 Robustness and sensitivity analyses

To improve transparency of the exploratory ROI-screening procedure, we quantified the number of ROI-parameter tests retained at each stage. In the GM, 98 of 512 ROI–parameter tests met the nominal ORP-association criterion, and 38 tests across 16 unique ROIs survived FDR correction. In the WM, 19 of 224 ROI–parameter tests met the nominal criterion, but none survived FDR correction. The paired tensor–kurtosis consistency criteria retained 19 ROI–parameter-pair combinations across 11 unique GM ROIs and 2 ROI–parameter-pair combinations across 2 unique WM ROIs. The screening flow and parameter-wise ROI counts are summarized in Supplementary Tables S3, S4.

LOSO analysis was performed to assess the stability of the ROI-level findings. For nominally significant ROI–parameter findings, the direction of the median ORP–diffusion association was preserved in all LOSO iterations. However, statistical retention varied across parameters. In GM, nominally significant AD, MD, and RD findings were retained in an average of 8.67, 6.96, and 7.30 of 9 iterations, respectively. In WM, nominally significant FA and KFA findings were retained in an average of 7.17 and 6.00 of 9 iterations, respectively. These results indicate that the main directional findings were stable, whereas the statistical significance of the individual ROIs remained sensitive to the modest sample size. Detailed LOSO results are provided in Supplementary Table S5.

We also characterized the first principal component used for global covariance control. PC1 explained 24.6%–45.1% of the variance in GM and 22.0%–37.0% in WM across diffusion parameters. PC1–ORP associations were modest in the GM, with absolute Spearman correlations ranging from 0.18 to 0.32, and weaker in WM, with absolute correlations ranging from 0.01 to 0.16. These findings indicate that PC1 captured substantial shared variance and may include sleep-depth-related physiological variance, particularly in the GM. Therefore, PC1-controlled analyses were interpreted as conservative global-covariance-controlled analyses rather than as simple removal of noise. The PC1 variance and PC1–ORP associations are summarized in Supplementary Table S6.

Network-level sensitivity analyses showed that the edge-level results depended on the thresholding strategy. The Q-based thresholding rule produced different network densities across parameters, and fixed-threshold analyses showed that the number of recurrent edges decreased as the absolute-correlation threshold became more stringent. In GM, recurrent edges across the six core tensor–kurtosis parameters decreased from 440 at |r| ≥ 0.20 to 71 at |r| ≥ 0.50. In WM, recurrent edges decreased from 15 at |r| ≥ 0.20 to 0 at |r| ≥ 0.45. These findings indicate threshold dependence, particularly for edge-level results, while also showing that the recurrent GM edge structure was not eliminated across a range of thresholds. Network thresholding and sensitivity analyses are summarized in Supplementary Tables S7, S8.

Finally, we performed a vascular-territory base-rate analysis to evaluate whether the distribution of sleep-depth-sensitive key edges exceeded the distribution expected from the number of possible ROI pairs. Among the 64 GM ROIs, 12 were assigned to ACA, 26 to MCA, and 26 to posterior-circulation categories. Four of the seven sleep-depth-sensitive key edges were MCA–MCA pairs. This exceeded the all-pair anatomical base rate; however, MCA enrichment was not significant when conditioned on same-territory edges. Therefore, the vascular-territory distribution was interpreted descriptively rather than as definitive evidence of MCA-specific enrichment. The vascular-territory base-rate analysis is provided in Supplementary Table S9.

## 4 Discussion

The present study investigated whether the diffusion tensor and diffusion kurtosis metrics vary with sleep depth in a manner potentially relevant to glymphatic physiology. To examine this relationship across repeated MRI acquisitions, we used ORP, an EEG-derived continuous measure of arousal propensity and sleep depth that reflects within-stage variation (Younes et al., 2015). The reconstructed ORP showed the expected separation across sleep stages, temporal correspondence with scorer-defined stage transitions, and high discrimination of wake versus non-wake epochs (area under the curve [AUC] = 0.931; Figure 2). In the MRI cohort, scan-wise ORP was characterized using 72 post-acquisition EEG segments corresponding to eight diffusion acquisitions in each of the nine participants. Averaging ORP across consecutive 30-s intervals substantially reduced its native 3-s variability, while its inverse association with relative delta power provided an internal EEG-based consistency check. However, temporal averaging may attenuate brief arousals and rapid state transitions, and post-acquisition EEG assumes relative stability of sleep depth between diffusion imaging and EEG measurement. Scan-wise ORP should therefore be interpreted as an estimate of the predominant, rather than invariant, sleep depth associated with each diffusion acquisition. Collectively, these findings provided a rationale for using reconstructed ORP in the subsequent ROI-level and network analyses.

After identifying significant ORP–diffusion associations, we focused on the ROIs that were retained across matched tensor–kurtosis pairs. In WM, decreases in both FA and KFA with increasing sleep depth suggest a reduced directional dependence of diffusion properties in the left corticospinal tract and sagittal stratum. The absence of corresponding diffusivity increases argues against a simple model of uniform ECS expansion and suggests that changes in directional water organization or compartmental signal contributions may be involved. In the cerebellar vermis, lower MD and RD, accompanied by higher MK and RK, indicate reduced water mobility and greater diffusional non-Gaussianity with deeper sleep, consistent with the involvement of the cerebellum in sleep-related physiology (Canto et al., 2017). Given that the observed diffusion changes occurred within approximately the first hour after sleep onset, they are more likely to reflect transient region-specific water redistribution than fixed structural remodeling. However, the present dMRI measures cannot identify the tissue or fluid compartments involved, and the WM findings require cautious interpretation because they did not survive FDR correction. In the GM, diffusion tensor and kurtosis parameters showed opposite directions of change with deeper sleep: AD, MD, and RD generally decreased, whereas AK, MK, and RK increased. This paired pattern suggests reduced water mobility accompanied by greater diffusional non-Gaussianity or microenvironmental heterogeneity within the same ROIs. It is not readily explained by a simple model of uniform ECS expansion, which is generally expected to increase diffusivity and potentially reduce kurtosis. Demiral et al. (2019) reported that sleep increased slow ADC in the cerebellum and left temporal pole, consistent with ECS expansion, but decreased fast ADC in the thalamus, insula, parahippocampus, and striatal regions. Because the fast ADC component may include contributions from perfusion-related signal and perivascular or convective fluid motion, its reduction is consistent with the decreased diffusivity observed in the present study. Additional evidence from sodium MRI suggests that the extracellular volume fraction in GM may decrease during deep NREM sleep (Watson et al., 2025), which is also consistent with the reduced diffusivity and increased kurtosis observed here. Because the observed changes occurred within approximately the first hour after sleep onset, they may be more consistent with transient, region-dependent redistribution or exchange of water among tissue and fluid compartments than with fixed structural remodeling. One possible, although speculative, explanation is a temporary regional mismatch between perivenous ISF clearance and periarterial CSF influx during early sleep. If local ISF clearance is not immediately balanced by the influx or exchange of CSF-derived water, the relative contribution of mobile extracellular water could decrease in some GM regions, increasing the relative contribution of more restricted and heterogeneous tissue water to the diffusion signal. However, such a temporal imbalance is not explicitly predicted by the conventional glymphatic model, and the present tensor and kurtosis measures cannot identify the compartments involved or determine the direction and temporal sequence of fluid transport. The GM findings should therefore be interpreted as coordinated sleep-depth-associated changes in diffusion properties, with an early imbalance between fluid influx and efflux considered a hypothesis-generating explanation rather than direct evidence of ECS expansion or glymphatic transport.

ROIs showing consistent ORP–diffusion parameter associations were primarily located in the frontocentral sensorimotor cortex and extended into the medial frontal and cingulate cortices. These regions correspond to canonical NREM physiology, in which slow-wave activity is generated and propagates through frontocentral and medial cortical territories (Massimini et al., 2004; Murphy et al., 2009; Nir et al., 2011). This spatial correspondence indicates that GM changes are not randomly distributed across the brain. However, although ROI analysis identifies where these changes occur, it does not determine whether they reflect independent local effects or coordinated interregional changes. Because these effects were observed in specific cortical regions, we examined whether they were coordinated across regions at the network level.

In addition to the ROI-level findings, diffusion parameter changes across repeated acquisitions were analyzed as ROI correlation networks. In these networks, edges represent temporal correlations between ROIs rather than anatomical connectivity. Graph-theoretical analysis suggested modest small-world-like organization across parameters (Table 2), consistent with locally clustered networks that are efficient compared with matched random networks. SAH nodes, operationally defined as high-strength hubs that also showed ORP–diffusion associations, were located mainly in sensorimotor and adjacent parietal regions (Table 5). This distribution identifies candidate regions in which sleep-depth sensitivity and network centrality co-occurred. This finding indicates that sleep-related diffusion changes are present in specific regions and are preferentially expressed in regions occupying central positions within the network.

**Table 5:** Sleep-associated hub nodes across diffusion parameters in WM and GM.

Tissue	Diffusion parameter	SAH nodes	Strength (z-score)
GM	AD	Precentral_L	1.4242
		Parietal_Inf_L	1.4070
		Postcentral_L	1.3685
		Temporal_Sup_L	1.3253
	AK	Precentral_L	2.0790
		Postcentral_L	2.0484
		Parietal_Sup_L	1.9258
		Paracentral_Lobule_L	1.7620
		Supp_Motor_Area_L	1.7169
	MD	Parietal_Inf_L	1.5546
		Temporal_Sup_L	1.3712
		Precentral_L	1.3606
		Postcentral_L	1.3545
	MK	Precentral_L	1.6355
		Paracentral_Lobule_L	1.5936
		Postcentral_L	1.5457
	RD	Parietal_Inf_L	1.6795
		Postcentral_L	1.5730
		SupraMarginal_L	1.3695
		Precentral_L	1.3529
		Temporal_Sup_L	1.3338
	RK	Parietal_Inf_L	2.2377
		Parietal_Sup_L	2.0480
		Precentral_L	1.8857
		Temporal_Sup_L	1.5024
	KFA	Temporal_Inf_L	1.8426
WM	FA	Posterior thalamic radiation_L	2.2386
		Inferior fronto-occipital fasciculus_L	1.2879
	KFA	Sagittal stratum_L	1.3842

ROIs with nodal strength exceeding z > 1.28 within each parameter-specific positive network were defined as hub nodes. These were intersected with ROIs that showed significant ORP–diffusion associations.

Key edges were defined as ROI pairs showing consistent correlations across multiple diffusion parameter networks after controlling for PC1. Of these, seven no longer met the six-network recurrence criterion after adjustment for sleep depth. These were classified as ORP-dependent, indicating sensitivity to sleep depth. Four of the seven ORP-dependent edges were located within the MCA territory, linking lateral frontal, insular/opercular, and temporoparietal cortical regions. These edges spanned multiple lateral cortical regions and were not limited to a single adjacent ROI pair. Thus, their correlations cannot be explained solely by anatomical proximity. ROIs within the same vascular territory may experience similar vascular and perivascular conditions, including related perfusion dynamics and neurovascular regulation. These shared conditions may produce similar diffusion parameter changes without requiring direct anatomical connectivity. This interpretation is relevant to glymphatic physiology, in which perivascular and vascular dynamics may influence sleep-related diffusion signals (Iliff et al., 2012; Xie et al., 2013). Two additional edges were located within VBA, linking cerebellar and vermian regions. These edges involved anatomically adjacent regions and may partly reflect spatial proximity or shared local tissue properties. The remaining edge was a CT connection between the paracentral lobule and precentral gyrus across vascular territories. This edge involved functionally related sensorimotor regions and indicates that sleep-related diffusion correlations are not restricted to a single vascular territory. However, it should not be interpreted as evidence of direct anatomical or functional connectivity, as it may reflect shared physiological influences not fully accounted for by PC1 and ORP adjustment.

However, the vascular-territory distribution should be interpreted descriptively. The MCA territory contains a relatively large number of possible ROI pairs, and the base-rate analysis showed that the MCA–MCA edge proportion exceeded the all-pair anatomical base rate but was not significant when conditioned on same-territory edges. Therefore, these findings should not be interpreted as definitive evidence of MCA-specific enrichment.

From this perspective, the correlation network provides a framework for characterizing sleep-related diffusion changes across distributed left-hemisphere and midline ROIs. Future studies should extend this framework to include conditions associated with pathological alterations in glymphatic function, such as sleep disorders, aging, and neurodegenerative diseases. In clinical settings, network-level features may help identify changes in physiological organization that are not apparent at the regional level. For example, alterations in the spatial distribution of interregional correlations, changes in network topology, or disruption of the relationship between sleep depth and diffusion parameter changes may reflect abnormal physiological coordination during sleep. In the future, integration with complementary imaging methods, including direct measurement of CSF flow, perfusion-sensitive acquisitions, and compartment-specific diffusion modeling, will be necessary to determine how these network features relate to underlying glymphatic processes. These approaches may help establish whether network-level diffusion metrics can serve as markers of altered physiological organization rather than merely reflecting regional abnormalities.

Several limitations should be considered. First, ORP was derived from the 90-s gradient-free EEG segment immediately following each diffusion acquisition, rather than during diffusion encoding itself, introducing temporal uncertainty when relating sleep depth to diffusion parameters from the preceding acquisition. Although summarization across three consecutive 30-s intervals reduced within-segment ORP variability, continuous artifact-suppressed EEG during diffusion encoding would allow more precise temporal alignment. Second, sleep was pharmacologically facilitated, and sleep-promoting medication may alter sleep architecture, neural activity, vascular dynamics, and fluid-related physiology. Because the study did not include a drug-free sleep condition, the effects of increasing sleep depth cannot be fully separated from medication-related effects, and the findings may not generalize directly to natural sleep. Third, ORP was derived from central EEG channels (C3 and C4), which may not fully capture frontal sleep features or the spatial heterogeneity of slow-wave activity. Fourth, the cohort was modest in size and demographically restricted, and the analyses were limited to the left hemisphere and midline regions because of EEG-related artifacts in the right hemisphere. Although LOSO analysis indicated directional stability of the main ROI findings, variation in statistical retention suggests that individual ROI- and edge-level results remain sensitive to sample size. Fifth, the initial ROI screening included nominal associations, particularly in WM, where no ROI survived FDR correction; the WM findings should therefore be interpreted as exploratory. Sixth, PC1 adjustment was used as a conservative control for global covariance, but PC1 explained substantial shared variance and may have included sleep-depth-related physiological variation. Seventh, the network findings were threshold-dependent, and the identified ORP-dependent edges should be considered candidate sleep-depth-sensitive associations rather than definitive network biomarkers. Their vascular-territory distribution should also be interpreted descriptively because the apparent concentration within the MCA territory was no longer significant after accounting for the base rate of same-territory edges. Finally, diffusion tensor and kurtosis parameters reflect the combined effects of tissue microenvironment, vascular dynamics, and fluid-related processes. ROI correlation networks quantify interregional covariation but do not establish directionality, anatomical connectivity, glymphatic transport, CSF–ISF exchange, or solute clearance. The observed patterns should therefore be interpreted as coordinated sleep-depth-associated changes in diffusion properties that may be relevant to glymphatic physiology, rather than as direct evidence of glymphatic transport or fluid movement.

## 5 Conclusion

Using simultaneous EEG and repeated multishell dMRI, we examined variations in brain diffusion parameters associated with sleep depth in humans. Deeper sleep was associated with distinct diffusion parameter changes in both the WM and GM. In WM, deeper sleep was associated with reduced diffusion anisotropy and kurtosis anisotropy, as reflected by decreased FA and KFA, respectively, in the left corticospinal tract and sagittal stratum. In GM, deeper sleep was associated with decreases in AD, MD, and RD and increases in AK, MK, and RK, indicating reduced diffusivity and greater diffusional non-Gaussianity or microenvironmental heterogeneity. Consistent with the spatial organization of NREM slow-wave activity, these changes were concentrated in the frontocentral sensorimotor and medial frontal–cingulate regions. Because the changes were observed within approximately the first hour after sleep onset, they may reflect transient, region-dependent water redistribution rather than fixed structural remodeling. At the network level, SAH nodes identified candidate regions in which sleep-depth sensitivity and network centrality co-occurred. After adjustment for global covariance and evaluation of ORP dependence, seven interregional correlations were identified as candidate sleep-depth-sensitive edges. Their vascular-territory distribution included MCA–MCA and posterior-circulation/VBA pairs, as well as a single cross-territory sensorimotor edge, but should be interpreted descriptively. Together, these findings indicate that sleep-related diffusion parameter changes are region-specific and organized as coordinated interregional covariation patterns. This study provides an exploratory framework for investigating sleep-depth-associated diffusion patterns potentially relevant to glymphatic physiology, but the findings do not establish ECS expansion or glymphatic transport as the underlying mechanism. Future studies incorporating larger cohorts undergoing natural sleep, EEG acquired concurrently with diffusion imaging, and complementary measures of perfusion, CSF dynamics, and tissue water compartments are required to clarify the underlying physiological mechanisms.

## Data Availability

The data supporting the findings of this study are available from the corresponding author upon reasonable request, subject to institutional and ethical restrictions.

## Glossary

AAL: automated anatomical labeling
ACA: anterior cerebral artery
ADC: apparent diffusion coefficient
AD: axial diffusivity
AK: axial kurtosis
AQP4: aquaporin-4
BH-FDR: Benjamini–Hochberg false discovery rate
CSF: cerebrospinal fluid
CT: cross-territory
DKI: diffusion kurtosis imaging
dMRI: diffusion magnetic resonance imaging
DTI: diffusion tensor imaging
DTI-ALPS: diffusion tensor image analysis along the perivascular space
ECS: extracellular space
EEG: electroencephalography
FA: fractional anisotropy
GM: gray matter
GS: glymphatic system
ISF: interstitial fluid
KFA: kurtosis fractional anisotropy
MCA: middle cerebral artery
MD: mean diffusivity
MK: mean kurtosis
MRI: magnetic resonance imaging
NREM: non-rapid eye movement
ORP: odds ratio product
PC1: first principal component
RD: radial diffusivity
RK: radial kurtosis
ROI: region of interest
SAH: sleep-associated hub
SHHS: Sleep Heart Health Study
ST: same-territory
VBA: vertebrobasilar/posterior circulation
WM: white matter
