# Supplementary material for: Regional and network patterns of sleep-depth-associated brain diffusion changes: an EEG-informed multishell diffusion MRI study
Source: Front Neurosci. 2026 Sep 15;20:1889651. doi: 10.3389/fnins.2026.1889651 (PMC13619960; doi:10.3389/fnins.2026.1889651)
Supplement: Supplementary file 1 [file Data_Sheet_1.docx]

Supplementary Material

# 1 Supplementary Figures and Tables

## 1.1 Supplementary Figures

**Supplementary Figure 1.** Modularity results for (a) WM and (b) GM.

(a)

(b)

**Supplementary Figure 2.** Parameter-specific ROI correlation matrices across WM and GM. (a–h) Group-averaged Spearman correlation matrices for diffusion tensor and diffusion kurtosis parameters in WM: AD, FA, AK, KFA, MD, RD, MK, and RK. (i–p) Corresponding matrices in GM.

**Supplementary Figure 3.** Full left-hemisphere and midline ROI network visualizations for (a–b) WM and (c–d) GM.(a)

(b)(c)(d)

## 1.2 Supplementary Tables

**Supplementary Table 1.** Complete listing of all ROIs exhibiting significant ORP–diffusion or ORP–kurtosis associations prior to enforcing paired-parameter concordance criteria**.**

| **Tissue** | **Diffusion parameter** | **ROI** | **Spearman correlation (median)** | ***p*-value** |
| --- | --- | --- | --- | --- |
| WM | AD | Fornix | 0.2667 | 0.0391 |
| WM | FA | Cerebral peduncle L | 0.3000 | 0.0273 |
| WM | FA | Corticospinal tract L | 0.3333 | 0.0195 |
| WM | FA | Inferior fronto-occipital fasciculus L | 0.2833 | 0.0273 |
| WM | FA | Pontine crossing tract | 0.4770 | 0.0039 |
| WM | FA | Posterior thalamic radiation L | 0.3000 | 0.0117 |
| WM | FA | Sagittal stratum L | 0.4333 | 0.0195 |
| WM | KFA | Corticospinal tract L | 0.3000 | 0.0078 |
| WM | KFA | Fornix (cres) / Stria terminalis L | 0.2500 | 0.0039 |
| WM | KFA | Sagittal stratum L | 0.4167 | 0.0195 |
| WM | MD | Fornix | 0.4500 | 0.0357 |
| WM | MD | Inferior fronto-occipital fasciculus L | -0.2833 | 0.0195 |
| WM | MK | Genu of corpus callosum | -0.3500 | 0.0039 |
| WM | MK | Posterior corona radiata L | 0.2500 | 0.0273 |
| WM | RD | Fornix | 0.2833 | 0.0195 |
| WM | RD | Fornix (cres) / Stria terminalis L | -0.3167 | 0.0195 |
| WM | RD | Inferior fronto-occipital fasciculus L | -0.3096 | 0.0273 |
| WM | RK | Body of corpus callosum | -0.4167 | 0.0195 |
| WM | RK | Fornix | -0.3333 | 0.0195 |
| GM | AD | Calcarine_L | 0.4167 | 0.0039 |
| GM | AD | Caudate_L | 0.4833 | 0.0078 |
| GM | AD | Cerebelum_7b_L | -0.3333 | 0.0273 |
| GM | AD | Cingulate_Ant_L | 0.3333 | 0.0195 |
| GM | AD | Cingulate_Mid_L | 0.4167 | 0.0039 |
| GM | AD | Cuneus_L | 0.3500 | 0.0195 |
| GM | AD | Frontal_Mid_2_L | 0.4667 | 0.0039 |
| GM | AD | Frontal_Sup_2_L | 0.5500 | 0.0039 |
| GM | AD | Frontal_Sup_Medial_L | 0.5000 | 0.0039 |
| GM | AD | Hippocampus_L | 0.2667 | 0.0173 |
| GM | AD | Occipital_Mid_L | 0.3167 | 0.0195 |
| GM | AD | Paracentral_Lobule_L | 0.4000 | 0.0039 |
| GM | AD | Parietal_Inf_L | 0.3333 | 0.0117 |
| GM | AD | Postcentral_L | 0.3667 | 0.0039 |
| GM | AD | Precentral_L | 0.4833 | 0.0039 |
| GM | AD | Supp_Motor_Area_L | 0.4167 | 0.0039 |
| GM | AD | Temporal_Pole_Mid_L | -0.4000 | 0.0117 |
| GM | AD | Temporal_Sup_L | 0.3167 | 0.0078 |
| GM | AK | Cerebelum_Crus2_L | 0.2667 | 0.0078 |
| GM | AK | Frontal_Mid_2_L | -0.3000 | 0.0078 |
| GM | AK | Frontal_Sup_2_L | -0.3667 | 0.0078 |
| GM | AK | Paracentral_Lobule_L | -0.2833 | 0.0039 |
| GM | AK | Parietal_Sup_L | -0.2667 | 0.0391 |
| GM | AK | Postcentral_L | -0.3000 | 0.0195 |
| GM | AK | Precentral_L | -0.2500 | 0.0078 |
| GM | AK | Supp_Motor_Area_L | -0.3167 | 0.0039 |
| GM | AK | Vermis_3 | -0.3333 | 0.0039 |
| GM | FA | Calcarine_L | 0.3333 | 0.0195 |
| GM | FA | Insula_L | 0.3180 | 0.0195 |
| GM | FA | OFClat_L | 0.4268 | 0.0195 |
| GM | FA | Putamen_L | 0.3167 | 0.0391 |
| GM | KFA | Frontal_Med_Orb_L | 0.2500 | 0.0391 |
| GM | KFA | OFClat_L | 0.3667 | 0.0039 |
| GM | KFA | Putamen_L | 0.2500 | 0.0499 |
| GM | KFA | Rolandic_Oper_L | 0.3180 | 0.0117 |
| GM | KFA | Temporal_Inf_L | 0.3833 | 0.0195 |
| GM | MD | Calcarine_L | 0.2833 | 0.0039 |
| GM | MD | Caudate_L | 0.5000 | 0.0078 |
| GM | MD | Cerebelum_7b_L | -0.2500 | 0.0273 |
| GM | MD | Cerebelum_Crus2_L | 0.3000 | 0.0195 |
| GM | MD | Cingulate_Ant_L | 0.4000 | 0.0078 |
| GM | MD | Cingulate_Mid_L | 0.4268 | 0.0117 |
| GM | MD | Cingulate_Post_L | 0.3500 | 0.0391 |
| GM | MD | Cuneus_L | 0.4000 | 0.0391 |
| GM | MD | Frontal_Inf_Oper_L | 0.2833 | 0.0273 |
| GM | MD | Frontal_Inf_Tri_L | 0.3167 | 0.0273 |
| GM | MD | Frontal_Mid_2_L | 0.5000 | 0.0039 |
| GM | MD | Frontal_Sup_2_L | 0.5500 | 0.0039 |
| GM | MD | Frontal_Sup_Medial_L | 0.4833 | 0.0039 |
| GM | MD | Hippocampus_L | 0.2500 | 0.0391 |
| GM | MD | Pallidum_L | -0.3500 | 0.0352 |
| GM | MD | Paracentral_Lobule_L | 0.4000 | 0.0117 |
| GM | MD | Parietal_Inf_L | 0.4167 | 0.0251 |
| GM | MD | Postcentral_L | 0.3833 | 0.0117 |
| GM | MD | Precentral_L | 0.4667 | 0.0039 |
| GM | MD | Supp_Motor_Area_L | 0.4833 | 0.0039 |
| GM | MD | Temporal_Pole_Mid_L | -0.3766 | 0.0039 |
| GM | MD | Temporal_Sup_L | 0.4833 | 0.0078 |
| GM | MD | Vermis_3 | 0.4167 | 0.0078 |
| GM | MK | Amygdala_L | -0.3500 | 0.0195 |
| GM | MK | Frontal_Med_Orb_L | -0.4167 | 0.0273 |
| GM | MK | Frontal_Mid_2_L | -0.3667 | 0.0039 |
| GM | MK | Frontal_Sup_2_L | -0.3833 | 0.0039 |
| GM | MK | Paracentral_Lobule_L | -0.2667 | 0.0078 |
| GM | MK | Postcentral_L | -0.3000 | 0.0117 |
| GM | MK | Precentral_L | -0.3000 | 0.0116 |
| GM | MK | Vermis_3 | -0.3667 | 0.0039 |
| GM | RD | Calcarine_L | 0.2500 | 0.0117 |
| GM | RD | Caudate_L | 0.4167 | 0.0078 |
| GM | RD | Cerebelum_7b_L | -0.3000 | 0.0273 |
| GM | RD | Cerebelum_8_L | -0.2833 | 0.0273 |
| GM | RD | Cerebelum_Crus2_L | 0.3000 | 0.0116 |
| GM | RD | Cingulate_Ant_L | 0.4333 | 0.0078 |
| GM | RD | Cingulate_Mid_L | 0.4167 | 0.0117 |
| GM | RD | Cingulate_Post_L | 0.3333 | 0.0273 |
| GM | RD | Frontal_Inf_Oper_L | 0.2594 | 0.0391 |
| GM | RD | Frontal_Inf_Tri_L | 0.4500 | 0.0195 |
| GM | RD | Frontal_Mid_2_L | 0.4333 | 0.0039 |
| GM | RD | Frontal_Sup_2_L | 0.5500 | 0.0039 |
| GM | RD | Frontal_Sup_Medial_L | 0.4519 | 0.0078 |
| GM | RD | OFClat_L | -0.3167 | 0.0391 |
| GM | RD | Paracentral_Lobule_L | 0.3500 | 0.0117 |
| GM | RD | Parietal_Inf_L | 0.3667 | 0.0391 |
| GM | RD | Postcentral_L | 0.3833 | 0.0117 |
| GM | RD | Precentral_L | 0.5000 | 0.0039 |
| GM | RD | Supp_Motor_Area_L | 0.4833 | 0.0039 |
| GM | RD | SupraMarginal_L | 0.4167 | 0.0173 |
| GM | RD | Temporal_Pole_Mid_L | -0.3833 | 0.0039 |
| GM | RD | Temporal_Sup_L | 0.4686 | 0.0078 |
| GM | RD | Vermis_3 | 0.4500 | 0.0078 |
| GM | RK | Angular_L | -0.2833 | 0.0298 |
| GM | RK | Frontal_Med_Orb_L | -0.2500 | 0.0273 |
| GM | RK | Frontal_Mid_2_L | -0.3833 | 0.0039 |
| GM | RK | Frontal_Sup_2_L | -0.4500 | 0.0039 |
| GM | RK | Parietal_Inf_L | -0.3000 | 0.0039 |
| GM | RK | Parietal_Sup_L | -0.3000 | 0.0195 |
| GM | RK | Precentral_L | -0.3500 | 0.0078 |
| GM | RK | SupraMarginal_L | -0.4667 | 0.0173 |
| GM | RK | Temporal_Sup_L | -0.3500 | 0.0273 |
| GM | RK | Vermis_3 | -0.3167 | 0.0117 |

**Supplementary Table 2.** Full list of 36 recurrent multi-parameter key edges and vascular-territory classification.

| **ROI 1** | **ROI 2** | **Territory 1** | **Territory 2** | **ST/CT** |
| --- | --- | --- | --- | --- |
| Vermis_4_5 | Vermis_6 | VBA | VBA | ST |
| Temporal_Inf_L | Temporal_Mid_L | MCA | MCA | ST |
| Postcentral_L | Precentral_L | MCA | MCA | ST |
| Paracentral_Lobule_L | Precuneus_L | ACA | ACA | ST |
| Paracentral_Lobule_L | Supp_Motor_Area_L | ACA | ACA | ST |
| OFCmed_L | Rectus_L | ACA | ACA | ST |
| OFCant_L | OFClat_L | MCA | MCA | ST |
| Occipital_Inf_L | Temporal_Inf_L | MCA | MCA | ST |
| Occipital_Inf_L | Occipital_Mid_L | MCA | MCA | ST |
| Frontal_Mid_2_L | Precentral_L | MCA | MCA | ST |
| Frontal_Inf_Tri_L | Frontal_Mid_2_L | MCA | MCA | ST |
| Frontal_Inf_Orb_2_L | OFClat_L | MCA | MCA | ST |
| Frontal_Inf_Oper_L | Frontal_Inf_Tri_L | MCA | MCA | ST |
| Frontal_Inf_Oper_L | Precentral_L | MCA | MCA | ST |
| Cerebelum_7b_L | Cerebelum_Crus2_L | VBA | VBA | ST |
| Cerebelum_4_5_L | Cerebelum_6_L | VBA | VBA | ST |
| Calcarine_L | Cuneus_L | PCA | PCA | ST |
| Calcarine_L | Lingual_L | PCA | PCA | ST |
| Angular_L | Parietal_Inf_L | MCA | MCA | ST |
| Precentral_L | Supp_Motor_Area_L | MCA | ACA | CT |
| Parietal_Sup_L | Postcentral_L | ACA | MCA | CT |
| Parietal_Inf_L | Parietal_Sup_L | MCA | ACA | CT |
| Paracentral_Lobule_L | Postcentral_L | ACA | MCA | CT |
| OFCant_L | OFCmed_L | MCA | ACA | CT |
| Occipital_Mid_L | Occipital_Sup_L | MCA | PCA | CT |

**Supplementary Table 3.** Exploratory ROI-screening flow.

| **Tissue** | **Screening stage** | **Count** | **Number of unique ROIs** |
| --- | --- | --- | --- |
| GM | Total atlas-based ROI-parameter tests | 512 | 64 |
| GM | Nominal ORP-associated ROI-parameter tests (*p*<0.05, $\left\vert\rho\right\vert\geq0.25$) | 98 | 35 |
| GM | FDR-significant ROI-parameter tests (*q*<0.05, $\left\vert\rho\right\vert\geq0.25$) | 38 | 16 |
| GM | Nominal paired tensor-kurtosis ROI pairs (both pair members *p*<0.05) | 19 | 11 |
| GM | Nominal paired tensor-kurtosis ROI pairs with expected sign relation | 19 | 11 |
| WM | Total atlas-based ROI-parameter tests | 224 | 28 |
| WM | Nominal ORP-associated ROI-parameter tests (*p*<0.05, $\left\vert\rho\right\vert\geq0.25$) | 19 | 11 |
| WM | FDR-significant ROI-parameter tests (*q*<0.05, $\left\vert\rho\right\vert\geq0.25$) | 0 | 0 |
| WM | Nominal paired tensor-kurtosis ROI pairs (both pair members *p*<0.05) | 2 | 2 |
| WM | Nominal paired tensor-kurtosis ROI pairs with expected sign relation | 2 | 2 |

**Supplementary Table 4.** ROI-level ORP–diffusion association counts by tissue and diffusion parameter.

| **Tissue** | **Diffusion Parameter** | **Number of atlas-based ROIs** | **Number of nominally significant ROIs** | **Number of FDR-significant ROIs** | **Minimum uncorrected *p*-value** | **Minimum FDR-adjusted q value** | **Median of ROI-wise median Spearman ρ** |
| --- | --- | --- | --- | --- | --- | --- | --- |
| GM | AD | 64 | 18 | 12 | 0.004 | 0.028 | 0.208 |
| GM | AK | 64 | 8 | 0 | 0.004 | 0.071 | -0.100 |
| GM | FA | 64 | 4 | 0 | 0.020 | 0.389 | 0.058 |
| GM | KFA | 64 | 4 | 0 | 0.004 | 0.250 | 0.033 |
| GM | MD | 64 | 23 | 11 | 0.004 | 0.036 | 0.183 |
| GM | MK | 64 | 8 | 0 | 0.004 | 0.083 | -0.117 |
| GM | RD | 64 | 23 | 15 | 0.004 | 0.045 | 0.158 |
| GM | RK | 64 | 10 | 0 | 0.004 | 0.083 | -0.108 |
| WM | AD | 28 | 1 | 0 | 0.008 | 0.219 | -0.017 |
| WM | AK | 28 | 0 | 0 | 0.055 | 0.684 | 0.000 |
| WM | FA | 28 | 6 | 0 | 0.004 | 0.109 | 0.150 |
| WM | KFA | 28 | 3 | 0 | 0.004 | 0.109 | 0.146 |
| WM | MD | 28 | 2 | 0 | 0.027 | 0.416 | 0.008 |
| WM | MK | 28 | 2 | 0 | 0.004 | 0.109 | 0.025 |
| WM | RD | 28 | 3 | 0 | 0.008 | 0.182 | -0.063 |
| WM | RK | 28 | 2 | 0 | 0.020 | 0.255 | 0.000 |

**Supplementary Table 5.** Leave-one-subject-out robustness (LOSO) of ROI-level ORP–diffusion associations

| **Tissue** | **Diffusion Parameter** | **Number of ROIs** | **Mean number of LOSO iterations preserving direction** | **Mean number of LOSO iterations retaining nominal significance** | **Mean number of LOSO iterations retaining FDR significance** |
| --- | --- | --- | --- | --- | --- |
| GM | AD | 18 | 9 | 8.667 | 1.222 |
| GM | AK | 8 | 9 | 7.875 | 0.000 |
| GM | FA | 4 | 9 | 7.250 | 0.000 |
| GM | KFA | 4 | 9 | 7.500 | 0.000 |
| GM | MD | 23 | 9 | 6.957 | 0.565 |
| GM | MK | 8 | 9 | 7.500 | 0.000 |
| GM | RD | 23 | 9 | 7.304 | 0.522 |
| GM | RK | 10 | 9 | 6.800 | 0.000 |
| WM | AD | 1 | 9 | 2.000 | 0.000 |
| WM | AK | 6 | 9 | 7.167 | 0.000 |
| WM | FA | 3 | 9 | 6.000 | 0.000 |
| WM | KFA | 2 | 9 | 3.500 | 0.000 |
| WM | MD | 2 | 9 | 6.500 | 0.000 |
| WM | MK | 3 | 9 | 7.000 | 0.000 |
| WM | RD | 2 | 9 | 9.000 | 0.000 |
| WM | RK | 12 | 9 | 9.000 | 1.667 |

**Supplementary Table 6.** PC1 variance explained and PC1–ORP association

| **Tissue** | **Diffusion Parameter** | **PC1 variance explained (%)** | **PC1–ORP Spearman ρ** | **Absolute PC1–ORP \|ρ\|** | ***p*-value** |
| --- | --- | --- | --- | --- | --- |
| GM | AD | 35.7 | -0.181 | 0.181 | 0.105 |
| GM | AK | 45.1 | 0.299 | 0.299 | 0.007 |
| GM | FA | 24.6 | 0.249 | 0.249 | 0.025 |
| GM | KFA | 36.9 | 0.321 | 0.321 | 0.004 |
| GM | MD | 35 | -0.194 | 0.194 | 0.084 |
| GM | MK | 39.6 | 0.311 | 0.311 | 0.005 |
| GM | RD | 34.3 | -0.180 | 0.18 | 0.109 |
| GM | RK | 32.4 | 0.291 | 0.291 | 0.008 |
| WM | AD | 28.6 | -0.073 | 0.073 | 0.516 |
| WM | AK | 33 | 0.163 | 0.163 | 0.146 |
| WM | FA | 25.7 | 0.078 | 0.078 | 0.492 |
| WM | KFA | 22 | 0.125 | 0.125 | 0.265 |
| WM | MD | 29.3 | -0.008 | 0.008 | 0.940 |
| WM | MK | 37 | 0.084 | 0.084 | 0.458 |
| WM | RD | 25.3 | 0.049 | 0.049 | 0.662 |
| WM | RK | 27.1 | -0.007 | 0.007 | 0.950 |

**Supplementary Table 7.** Network thresholding and core graph-theoretical summary.

| **Tissue** | **Diffusion Parameter** | **Selected \|r\| threshold** | **Target Q** | **Positive hubs** | **Negative hubs** | **Mean retained edges** | **Mean density** | **Mean γ** | **Mean λ** | **Mean σ** |
| --- | --- | --- | --- | --- | --- | --- | --- | --- | --- | --- |
| GM | AD | 0.48 | 0.3 | 8 | 9 | 744.7 | 0.369 | 1.31 | 1.03 | 1.27 |
| GM | AK | 0.48 | 0.3 | 12 | 7 | 595.7 | 0.295 | 1.4 | 1.04 | 1.35 |
| GM | FA | 0.28 | 0.3 | 11 | 8 | 1027.2 | 0.51 | 1.08 | 1 | 1.08 |
| GM | KFA | 0.4 | 0.3 | 9 | 8 | 708.4 | 0.351 | 1.29 | 1.01 | 1.28 |
| GM | MD | 0.48 | 0.3 | 9 | 8 | 746.3 | 0.37 | 1.33 | 1.03 | 1.28 |
| GM | MK | 0.32 | 0.3 | 11 | 8 | 1085.4 | 0.538 | 1.09 | 1 | 1.09 |
| GM | RD | 0.46 | 0.3 | 11 | 6 | 769.1 | 0.382 | 1.33 | 1.03 | 1.29 |
| GM | RK | 0.1 | 0.3 | 12 | 13 | 1676.8 | 0.832 | 1 | 1 | 1 |
| WM | AD | 0.28 | 0.3 | 5 | 5 | 191.8 | 0.507 | 1.07 | 1 | 1.07 |
| WM | AK | 0.3 | 0.3 | 4 | 4 | 170.7 | 0.451 | 1.12 | 1 | 1.12 |
| WM | FA | 0.28 | 0.3 | 5 | 4 | 195.3 | 0.517 | 1.06 | 1 | 1.06 |
| WM | KFA | 0.26 | 0.3 | 2 | 4 | 204.2 | 0.54 | 1.05 | 1 | 1.05 |
| WM | MD | 0.28 | 0.3 | 6 | 5 | 198.7 | 0.526 | 1.07 | 1 | 1.07 |
| WM | MK | 0.32 | 0.3 | 5 | 3 | 178.2 | 0.471 | 1.14 | 1 | 1.13 |
| WM | RD | 0.28 | 0.3 | 6 | 4 | 194.7 | 0.515 | 1.08 | 1 | 1.08 |
| WM | RK | 0.34 | 0.3 | 6 | 4 | 162.1 | 0.429 | 1.17 | 1.01 | 1.16 |

**Supplementary Table 8.** Network sensitivity analyses with and without PC1 control and across fixed absolute-correlation thresholds.

| **Tissue** | **Fixed threshold** | **Edges recurrent across six core parameters** |
| --- | --- | --- |
| GM | 0.20 | 440 |
| GM | 0.25 | 327 |
| GM | 0.30 | 240 |
| GM | 0.35 | 189 |
| GM | 0.40 | 141 |
| GM | 0.45 | 95 |
| GM | 0.50 | 71 |
| WM | 0.20 | 15 |
| WM | 0.25 | 8 |
| WM | 0.30 | 5 |
| WM | 0.35 | 3 |
| WM | 0.40 | 1 |
| WM | 0.45 | 0 |
| WM | 0.50 | 0 |

**Supplementary Table 9.** Vascular-territory base-rate analysis for sleep-depth-sensitive key edges.

| **Tissue** | **Screening stage** |
| --- | --- |
| Total GM ROIs | 64 |
| ACA-assigned ROIs | 12 |
| MCA-assigned ROIs | 26 |
| Posterior-circulation ROIs | 26 |
| Possible MCA–MCA ROI pairs | 325 / 2016 |
| Observed sleep-depth-sensitive MCA–MCA edges | 4 / 7 |
| Observed VBA/posterior-circulation edges | 2 / 7 |
| Observed cross-territory ACA–MCA edge | 1 / 7 |
| All-pair MCA enrichment p value | 0.0155 |
| Same-territory conditional MCA enrichment p value | 0.2609 |

**Supplementary Table 10.** Stability of scan-wise ORP estimates and their association with relative delta power in dMRI-matched post-acquisition EEG segments.

| **Measure** | **Definition** | **N** | **Summary** | **IQR** | ***p*-value** |
| --- | --- | --- | --- | --- | --- |
| Segment duration | Duration of post-acquisition EEG segment, s | 72 | 85.85 | 83.00–89.00 | — |
| Scan-wise ORP | Mean ORP across three 30-s summaries | 72 | 0.280 | 0.202–0.457 | — |
| 3-s ORP SD | Within-segment SD of consecutive 3-s ORP estimates | 72 | 0.233 | 0.138–0.371 | — |
| 3-s ORP range | Within-segment range of consecutive 3-s ORP estimates | 72 | 1.051 | 0.589–1.419 | — |
| 30-s ORP SD | SD across three consecutive 30-s ORP summaries | 72 | 0.070 | 0.032–0.143 | — |
| 30-s ORP range | Range across three consecutive 30-s ORP summaries | 72 | 0.129 | 0.056–0.272 | — |
| Relative delta power | Relative power in the 0.5–4 Hz band | 72 | 0.700 | 0.533–0.791 | — |
| ORP–delta association, pooled | Spearman correlation across 72 segments | 72 | ρ = -0.515 | — | 3.73 × 10^-6^ |
| ORP–delta association, subject-wise | Median within-subject Spearman correlation | 9 | ρ = -0.333 | -0.810 to -0.214 | 0.0078 |

Note. Values were calculated from the 72 post-acquisition EEG segments corresponding to the eight sleep-state diffusion MRI acquisitions in nine participants. ORP was calculated from consecutive 3-s epochs and summarized as the mean of three consecutive 30-s interval summaries. Relative delta power was calculated as power in the conventional 0.5–4 Hz band normalized by total power in the 0.5–35 Hz range. For the subject-wise ORP–delta association, Spearman correlations were first calculated within each participant and then tested against zero using a Wilcoxon signed-rank test. ORP, odds ratio product; dMRI, diffusion MRI; IQR, interquartile range; SD, standard deviation.
